# Mouse Gonad Development in the Absence of the Pro-Ovary Factor WNT4 and the Pro-Testis Factor SOX9

**DOI:** 10.3390/cells9051103

**Published:** 2020-04-29

**Authors:** Furong Tang, Nainoa Richardson, Audrey Albina, Marie-Christine Chaboissier, Aitana Perea-Gomez

**Affiliations:** CNRS, Inserm, Institut de Biologie Valrose (iBV), Université Côte d’Azur, 06108 Nice, France; Furong.TANG@hotmail.com (F.T.); Nainoa.Richardson@ucsf.edu (N.R.); audrey.albina@etu.parisdescartes.fr (A.A.); Marie-Christine.CHABOISSIER@univ-cotedazur.fr (M.-C.C.)

**Keywords:** gonad development, sex determination, SOX9, WNT signaling, ovotestis

## Abstract

The transcription factors SRY and SOX9 and RSPO1/WNT4/β-Catenin signaling act as antagonistic pathways to drive testis and ovary development respectively, from a common gonadal primordium in mouse embryos. In this work, we took advantage of a double knockout mouse model to study gonadal development when *Sox9* and *Wnt4* are both mutated. We show that the XX gonad mutant for *Wnt4* or for both *Wnt4* and *Sox9* develop as ovotestes, demonstrating that ectopic SOX9 function is not required for the partial female-to-male sex reversal caused by a *Wnt4* mutation. *Sox9* deletion in XY gonads leads to ovarian development accompanied by ectopic WNT/β-catenin signaling. In XY *Sox9* mutant gonads, SRY-positive supporting precursors adopt a female-like identity and develop as pre-granulosa-like cells. This phenotype cannot be fully prevented by the deletion of *Wnt4* or *Rspo1*, indicating that SOX9 is required for the early determination of the male supporting cell identity independently of repressing RSPO1/WNT4/β-Catenin signaling. However, in XY *Sox9 Wnt4* double mutant gonads, pre-granulosa cells are not maintained, as they prematurely differentiate as mature granulosa cells and then trans-differentiate into Sertoli-like cells. Together, our results reveal the dynamics of the specific and independent actions of SOX9 and WNT4 during gonadal differentiation: SOX9 is essential in the testis for early specification of male-supporting cells whereas WNT4 functions in the ovary to maintain female-supporting cell identity and inhibit male-specific vascular and steroidogenic cell differentiation.

## 1. Introduction

In mammals, gonadal sex determination is a highly controlled developmental process leading to the formation of a testis or ovary from a common primordium present in XY and XX organisms at embryonic stages (reviewed by [1]). Sex hormones subsequently secreted by the testis or the ovary, promote the development of secondary sexual characteristics to maintain the sexual identity and fertility [1].

The male sex determining gene *Sry,* located on the Y-chromosome, is expressed in mouse XY gonads from embryonic day 10.5–12.5, or E10.5–E12.5 [2,3,4,5,6]. SRY activates the expression of another high-mobility group (HMG) box-family transcription factor, SOX9, which, in turn, regulates other genes required to establish the Sertoli cell lineage that will further orchestrate testis development [7,8,9,10]. XY *Sox9* mutant mice exhibit complete sex reversal and develop ovaries capable of producing oocytes that are chromosomally X or Y [11,12,13].

In the absence of Y chromosome, XX gonadal supporting cells differentiate as FOXL2-positive pre-granulosa cells and enter into mitotic arrest marked by the expression of cyclin-dependent kinase inhibitor CDKN1B/P27 [14,15]. Though FOXL2 is required to maintain granulosa cell identity in post-natal ovaries, this transcription factor is dispensable in the mouse ovary during embryonic stages [16,17]. In contrast, RSPO1/WNT4/β-Catenin signaling is required for embryonic ovarian development in both mice and human [18,19,20,21,22,23,24]. Mouse XX gonads harboring mutations in *Wnt4*, *Rspo1*, or *Ctnnb1* (encoding β-Catenin) progressively develop as ovotestes, with characteristics of testes and ovaries [18,19,20,21,25]. The development of the partially sex-reversed gonads has been characterized and involves pre-granulosa cells first exiting mitotic arrest and differentiating prematurely as mature granulosa cells expressing AMH in addition to FOXL2 [25,26]. Next, mature granulosa cells loose FOXL2 expression, trans-differentiate into SOX9 and AMH positive Sertoli-like cells and organize as testis cord-like structures around birth [18,25]. In addition, RSPO1/WNT4/β-Catenin deficient XX gonads develop a testis-like coelomic vessel at E12.5 due to ectopic migration of endothelial cells from the adjacent mesonephros [18,20,21,27]. Additionally, *Wnt4*, *Rspo1*, and *Ctnnb1* XX mutant gonads exhibit ectopic steroidogenic cells, which are absent in embryonic ovaries [18,19,20,21,28,29]. These cells produce testosterone and masculinize the XX genital tracts. Germ cells are depleted through apoptosis from E16.5 in *Wnt4* and *Ctnnb1* XX mutants [19,30,31] or by reduced proliferation from E12.5 in *Rspo1* XX mutants [32].

Single-cell RNA-seq analyses of developing gonads have identified an early supporting cell precursor population with similar transcriptional profiles in XY and XX mouse embryos [33]. Differentiation of testicular Sertoli cells and ovarian granulosa cells in testes and ovaries respectively requires activation of the male or female pathway and repression of the alternate genetic cascade. Indeed, it has been demonstrated that ectopic activation of WNT/β-Catenin signaling or FOXL2 in XY gonads results in down-regulation of SOX9 and is sufficient to induce ovarian development [34,35,36]. Conversely, transgenic expression of SRY and, thus, upregulation of SOX9 or, simply, transgenic expression of SOX9, in embryonic XX supporting cells can induce testicular development [37,38,39].

Studies in double mutant mice gonads have also supported the principle of antagonistic sex determination pathways. One example involves fibroblast growth factor 9 (FGF9), which, when bound to its receptor FGFR2c, activates *Sox9* expression in Sertoli cells to promote rapid expansion of the male supporting cell lineage throughout the developing testis [40,41]. Mutations in *Fgf9* or *Fgfr2/Fgfr2c* lead to reduced SOX9 expression and partial male-to-female sex reversal [40,41,42,43,44]. In XY *Fgf9 Wnt4*, *Fgfr2 Wnt4*, and *Fgfr2c Foxl2* double mutants, SOX9 expression and testicular differentiation are restored, indicating that FGF9 also functions to antagonize WNT4- and FOXL2-mediated repression of *Sox9* [41,45]. The outcome of mutating *Sox9* together with the female pathway components *Rspo1* or *Ctnnb1* has also been studied [26,46]. The gonads of both XX *Sox9 Rspo1* and XX *Sox9 Ctnnb1* double mutants develop as ovotestes, demonstrating that other factors besides SRY and SOX9 can drive Sertoli-like cell differentiation in *Rspo1* and *Ctnnb1* mutants [26,46]. In XY individuals, *Sox9 Ctnnb1* double mutant embryonic gonads develop as ovotestes [26] and *Sox9 Rspo1* mutant post-natal gonads develop as hypo-plastic testes [46]. These results indicate that although deletion of *Rspo1* or *Ctnnb1* can restore some aspects of testicular development in XY *Sox9* mutant gonads, complete testis differentiation requires SOX9 function, even when the female WNT/ß-Catenin pathway is impaired.

While the gonad outcome of XY and XX *Sox9* mutant mice also lacking *Rspo1* or *Ctnnb1* has been investigated, the gonad fate in *Sox9 Wnt4* double mutants has not yet been reported. Furthermore, the sequence of events leading to the appearance of testicular characteristics in XY *Sox9 Ctnnb1* and *XY Sox9 Rspo1* double mutant gonads are unknown. In this study, we report the generation and analysis of double mutants for *Sox9* and *Wnt4* at different time points during gonadal development. We show here that similar to XX *Sox9 Rspo1* and XX *Sox9 Ctnnb1* mutants, SOX9 function is not required for the masculinization of XX *Wnt4* mutant gonads. In addition, we demonstrate that loss of *Wnt4* does not prevent the specification of XY supporting cell progenitors as pre-granulosa-like cells in XY *Sox9* mutant gonads. However, in XY *Sox9 Wnt4* mutants, granulosa-like cells trans-differentiate into Sertoli-like cells, leading to ovotestes development near birth.

This work highlights the specific and sequential actions of SOX9 and WNT4 during gonadal differentiation: SOX9 is essential for early specification of male supporting cells whereas WNT4 is required to maintain female supporting cell identity and to inhibit male-specific vascular and steroidogenic cell differentiation in developing ovaries.

## 2. Materials and Methods

### 2.1. Mouse Strains and Genotyping

The experiments described herein were carried out in compliance with the relevant institutional and European animal welfare laws, guidelines and policies. These procedures were approved by the French ethics committee (Comité institutionnel d’Ethique pour l’Animal de Laboratoire, number NCE/2011-2112, approved 2011/06/22). All mouse lines were kept on a mixed 129/C57Bl6/J background. *Sf1-Cre^tg/0^*; *Sox9^flox/flox^*; *Wnt4^+/−^* females were crossed with *Sf1-Cre^tg/0^*; *Sox9^flox/+^*; *Wnt4^+/−^* males to obtain mutant embryos at different stages. Embryos were named controls (*Sf1-Cre^tg^*; *Sox9^flox/+^*; *Wnt4^+/−^*), *Sox9^cKO^* mutants (*Sf1-Cre^tg^; Sox9^flox/flox^*; *Wnt4^+/−^*), *Wnt4^KO^* mutants (*Sf1-Cre^tg^*; *Sox9^flox/+^*; *Wnt4^−/−^*), and *Sox9^cKO^ Wnt4^KO^* double mutants (*Sf1-Cre^tg^*; *Sox9^flox/flox^*; *Wnt4^−/−^*). The generation of *Sf1-Cre^tg/0^*; *Sox9^flox/flox^*; *Rspo1^−/−^* mice (*Sox9^cKO^ Rspo1^KO^*) has been previously described [46]. Genotypes of mice and embryos were determined using PCR assays on lysates from ear biopsies or tail tips as previously described [11,13,19,47]. The day when a vaginal plug was found was designated as embryonic day E0.5. E10.5–E12.5 embryos were staged by counting the number of tail somites (ts) with 8 ts corresponding to E10.5, 18 ts to E11.5 and 30 ts to E12.5 [5].

### 2.2. Immunofluorescence Staining

Sections (5 µm) of samples fixed in Bouin’s solution (HT10132, Sigma-Aldrich, Munich, Germany), Antigen fix (P10016, Diapath, Martinengo, Italy) or 4% (*w/v*) paraformaldehyde (PFA, 15710-S, EMS, Hatfield, PA, USA) were processed for immunofluorescence staining. Paraffin was removed in xylene baths, and sections were rehydrated in 100%, 90%, 70%, 40% ethanol (EtOH) and H_2_O. Antigen retrieval was obtained by pressure cooking in 10 mM sodium citrate pH 6, 0.05% TWEEN^®^ 20 (P1379, Sigma-Aldrich) for 10 min. Sections were blocked with 3% bovine serum albumin (BSA, A2153, Sigma-Aldrich), 10% inactivated normal donkey serum (NDS, D9663-10ML, Sigma-Aldrich), 0.1% TWEEN^®^ 20 in phosphate buffered saline (PBS) for 1 h at room temperature. Primary antibodies diluted in 3% BSA, 3% NDS, 0.1% TWEEN^®^ 20, PBS were incubated at 4 °C overnight. After three washes in 0.1% TWEEN^®^ 20, PBS, secondary antibodies diluted in PBS were incubated for 1 h at room temperature. After three washes in 0.1% TWEEN^®^ 20, PBS, slides were mounted with Vectashield Hardset solution containing DAPI (H-1500, Vector Laboratories, Peterborough, UK). Images were obtained on a motorized Axio Imager Z1 microscope (Zeiss, Oberkochen, Germany) coupled with an AxioCam MRm camera (Zeiss, Oberkochen, Germany) and processed with Fiji (Bethesda, MD, USA) and Adobe Photoshop (San Jose, CA, USA). Antibodies are listed in Table S1. At least three embryos of each genotype were analyzed for each marker.

### 2.3. RNAscope^®^ In Situ Hybridization

Gonads were dissected, fixed in 4% (*w/v*) PFA (15710-S, EMS) overnight at room temperature and embedded in paraffin. Sections (5 µm) were hybridized with *Wnt4* probe (Advanced Cell Diagnostics, Newark, NJ, USA). The RNAscope^®^ assay combined with immunofluorescence was performed according to the manufacturer’s instructions. Images were obtained on a LSM 710 confocal microscope (Zeiss) and processed with Fiji and Adobe Photoshop.

### 2.4. RNA Extraction and Quantitative PCR Analysis

Individuals gonads without mesonephros were dissected in PBS, snap-frozen in liquid nitrogen and kept at −80 °C. Total RNAs were extracted by RNeasy Micro Kit (74004, Qiagen, Manchester, UK) and reverse transcribed by M-MLV reverse transcriptase (M170A, Promega, Madison, WI, USA). Quantitative-PCR reactions were prepared in 10 μL with LightCycler^®^ 480 SYBR Green I Master (04887352001, Roche, Basel, Switzerland) and run in the LightCycler^®^ 480 System (05015278001, Roche), q-PCR primers are listed in Table S2. All biological replicates of different genotypes (*N* = 3–5) were run in the same plate and repeated at least twice. Relative gene expression of each gonad was calculated based on a standard curve from each run and normalized to the expression of the housekeeping gene *Shda*. Relative normalized gene expressions in all genotypes were compared to relative normalized gene expression in XX controls. Fold change in gene expression was obtained by dividing the normalized gene expression in gonads of a given genotype by the mean of the normalized gene expression in XX control gonads. Graphs show the individual values of relative normalized gene expression compared to XX control (dots), as well as the mean fold-change (bars) ± SEM.

## 3. Results

### 3.1. Efficient Genetic Deletion of Sox9 in Sox9^cKO^ Wnt4^KO^ Double Mutants

To generate *Sox9^cKO^ Wnt4^KO^* double mutants, we utilized a *Wnt4* mutant line (*Wnt4^KO^*) where deletion of exon 3 leads to a WNT4 loss-of-function [19], and a conditional *Sox9* mutant line, where deletion of *Sox9* exons 2 and 3 occurs in cells expressing the *Sf1-Cre* transgene (*Sox9^cKO^*) [13,47]. *Sf1-Cre* is active in the somatic cells of the gonad from E10.5, and is also expressed in the adrenal cortex, the spleen, the anterior pituitary and the ventromedial hypothalamus [47]. Previous work has established that deletion of *Sox9* by *Sf1-Cre* leads to ovarian development in XY mutant gonads, a phenotype that can be attributed to SOX9 loss-of-function in the somatic gonad independently of hypothalamus-pituitary function [13]. Quantitative PCR analysis at E12.5 and E14.5 confirmed that *Sox9* was also efficiently deleted in the gonad of single and double mutant embryos generated for this study (Figure S1).

### 3.2. Early Phenotypic Changes in XX Wnt4^KO^ Mutants are Independent of SOX9 Function

**Figure 1:**
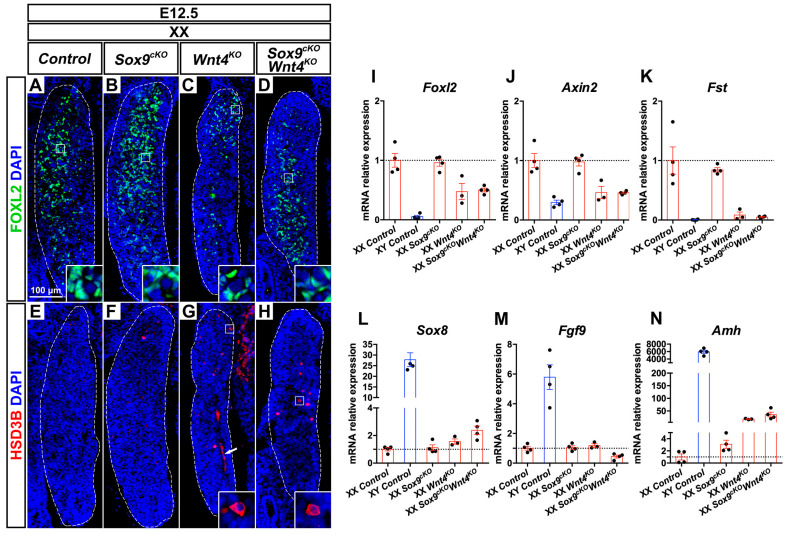
Early phenotypic changes in XX *Wnt4* mutants are independent of SOX9 function. (**A**–**D**) Immunodetection of the pre-granulosa cell marker FOXL2 (green) in E12.5 XX gonads of *Control* (**A**), *Sox9^cKO^* (**B**), *Wnt4^KO^* (**C**) and *Sox9^cKO^ Wnt4^KO^* (**D**) genotypes. (**E**–**H**) Immunodetection of the steroidogenic enzyme HSD3B (red) in E12.5 XX gonads of *Control* (**E**), *Sox9^cKO^* (**F**), *Wnt4^KO^* (**G**) and *Sox9^cKO^ Wnt4^KO^* (**H**) genotypes. (**I**–**N**) RT-quantitative PCR analysis of *Foxl2* (**I**), *Axin2* (**J**), *Follistatin* (*Fst*) (**K**), *Sox8* (**L**), *Fgf9* (**M**) and *Amh* (**N**) expression in E12.5 gonads of XX *Control*, XY *Control*, XX *Sox9^cKO^*, XX *Wnt^4KO^*, and XX *Sox9^cKO^ Wnt4^KO^* genotypes (*N* = 3–4 embryos for each genotype). Expression level in XX controls is 1. Graphs show individual values (dots), and the mean fold-change (bars) ± SEM. Nuclei labeled with DAPI are shown in blue. Magnification is the same in all panels. Scale bar = 100 μm. Gonads are outlined with broken white lines. White arrow in G indicates the coelomic vessel.

Given that *Sox9* is transiently up-regulated in XX *Wnt4^KO^* mutant ovaries between E11.5 and E12.0 [40], we aimed to determine the functional contribution of SOX9 in XX *Wnt4^KO^* mutant gonads. Thus, we compared XX *Sox9^cKO^ Wnt4^KO^* double mutant gonads at E12.5 and E14.5 with the respective single mutants and control gonads. At E12.5, XX control and XX *Sox9^cKO^* mutant gonads contain pre-granulosa cells with robust expression of the transcription factor FOXL2 (Figure 1A,B and Figure S2). In contrast, XX *Wnt4^KO^* mutants showed a marked reduction in FOXL2/*Foxl2* expression at E12.5 and E14.5 (Figure 1C,I and Figure S2). This observation is consistent with reports indicating that *Foxl2* expression is regulated in part by WNT/β-Catenin signaling [26,48,49]. Indeed, loss of *Wnt4* in XX embryos compromised WNT/β-Catenin activity, as shown by reduced expression of *Axin2* (Figure 1J and Figure S2), a downstream target of this pathway. In XX *Sox9^cKO^ Wnt4^KO^* double mutant gonads, FOXL2/*Foxl2* and *Axin2* expression was similarly reduced (Figure 1D,I–J and Figure S2), indicating that down-regulation of these factors does not depend on the presence of SOX9.

Next, we asked whether XX *Sox9^cKO^ Wnt4^KO^* gonads contained steroidogenic cells and developed a coelomic vessel, as in XY control gonads and XX *Wnt4^KO^* mutants [19]. XX controls lacked cells expressing the steroidogenic enzyme HSD3β, and 2 out of 3 XX *Sox9^cKO^* mutant gonads exhibited only rare HSD3β positive cells (Figure 1E–F). In contrast, these cells were found in all XX *Wnt4^KO^* and XX *Sox9^cKO^ Wnt4^KO^* mutant gonads (Figure 1G,H and Figure S2). Furthermore, XX *Wnt4^KO^* and *Sox9^cKO^ Wnt4^KO^* mutant gonads developed a coelomic vessel (Figure 1G, arrow), similar to XY control testes [27]. In testes, coelomic vessel formation is attributed to migration of endothelial cells from the mesonephros, which in the developing ovary is inhibited by Follistatin (*Fst*) [27,30]. Accordingly, we found that *Fst* expression was severely down-regulated in XX *Wnt4^KO^* and *Sox9^cKO^ Wnt4^KO^* mutant gonads at E12.5 (Figure 1K).

In our analyses, we did not detect up-regulation of *Sox9* and *Fgf9* in XX *Wnt4^KO^* mutant gonads (Figure 1M and Figure S1), as previously reported [40]. However, XX *Wnt4^KO^* and XX *Sox9^cKO^ Wnt4^KO^* mutant gonads exhibited an enrichment for transcripts expressed in Sertoli cells, such as *Sox8, Amh,* and *Dhh* at E12.5 and E14.5, with *Sox8* only being transiently expressed at E12.5 (Figure 1L,N and Figure S2).

Altogether, by analyzing XX *Sox9^cKO^ Wnt4^KO^* double mutant gonads, our data demonstrates that SOX9 function is not required for the reduction of WNT/β-catenin signaling and FOXL2 expression, the up-regulation of Sertoli cell markers, and the formation of ectopic vasculature and steroidogenic cells. We conclude that the establishment of early phenotypic changes in *Wnt4^KO^* XX mutants is independent of SOX9 function.

### 3.3. Late Phenotypic Changes in XX Wnt4^KO^ Mutants are Independent of SOX9 Function

**Figure 2:**
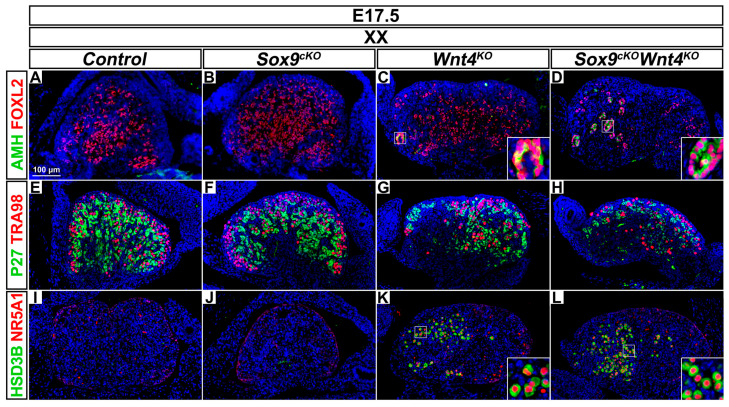
Abnormal ovarian development in XX *Wnt4* mutants is independent of SOX9 function. (**A**–**D**) Immunodetection of the differentiation marker AMH (green) and the pre-granulosa cell marker FOXL2 (red) in E17.5 XX gonads of *Control* (**A**), *Sox9^cKO^* (**B**), *Wnt4^KO^* (**C**) and *Sox9^cKO^ Wnt4^KO^* (**D**) genotypes. (**E**–**H**) Immunodetection of the quiescent cell marker P27 (green) and the germ cell marker TRA98 (red) in E17.5 XX gonads of *Control* (**E**), *Sox9^cKO^* (**F**), *Wnt4^KO^* (**G**) and *Sox9^cKO^ Wnt4^KO^* (**H**) genotypes. (**I**–**L**) Immunodetection of the steroidogenic enzyme HSD3β (green) and the steroidogenic transcription factor NR5A1 (red) in E17.5 XX gonads of *Control* (**I**), *Sox9^cKO^* (**J**), *Wnt4^KO^* (**K**), and *Sox9^cKO^ Wnt4^KO^* (**L**) genotypes. Nuclei labeled with DAPI are shown in blue. Magnification is the same in all panels. Scale bar = 100 μm.

We then focused on XX *Wnt4^KO^* mutant gonads developing without *Sox9* at E17.5. At this stage, a loss of fetal ovarian integrity is observed in XX *Wnt4^KO^* mutant gonads, since pre-granulosa cells differentiate prematurely [25]. Accordingly, at E17.5, FOXL2-positive pre-granulosa cells in the anterior part of XX *Wnt4^KO^* mutant gonads precociously exit their mitotic arrest, as indicated by down-regulation of P27 (CDKN1B) and prematurely differentiate as mature granulosa cells expressing AMH [25,50] (Figure 2C,G). Similarly, XX *Sox9^cKO^ Wnt4^KO^* double mutant gonads exhibited mature granulosa cells (Figure 2D,H). These results indicate that in XX *Wnt4^KO^* mutants, SOX9 function is dispensable for premature granulosa cell differentiation, a phenotype that precedes reprogramming as Sertoli-like cells [25]. However, by using immunostaining for the Sertoli marker DMRT1 [51], we did not observe Sertoli cells forming testis cords in XX *Wnt4^KO^* mutant and XX *Sox9^cKO^ Wnt4^KO^* double mutant gonads at E17.5 and P0.

Though the lethality of the *Wnt4^KO^* mutation after birth prevented further assessment of the role of SOX9 for the appearance of Sertoli-like cells in XX *Wnt4^KO^* mutants, additional characteristics associated with sex reversal were apparent. These include a loss of germ cells in the anterior region [30] and the ectopic presence of steroidogenic cells [19]. Here, we observed a reduction of the germ cells expressing the TRA98 antigen and an abundance of steroidogenic cells expressing HSD3β and NR5A1 in the anterior region of XX *Wnt4^KO^* and XX *Sox9^cKO^ Wnt4^KO^* mutant gonads at E17.5 in comparison to controls and XX *Sox9^cKO^* mutants (Figure 2E–L). Thus, our analyses at E17.5 demonstrate that premature granulosa cell differentiation, germ cell loss, and ectopic steroidogenic cell formation in XX *Wnt4^KO^* mutant gonads are independent of SOX9 function.

### 3.4. Wnt4 Expression is Up-Regulated in XY Sox9^cKO^ Mutant Gonads

**Figure 3:**
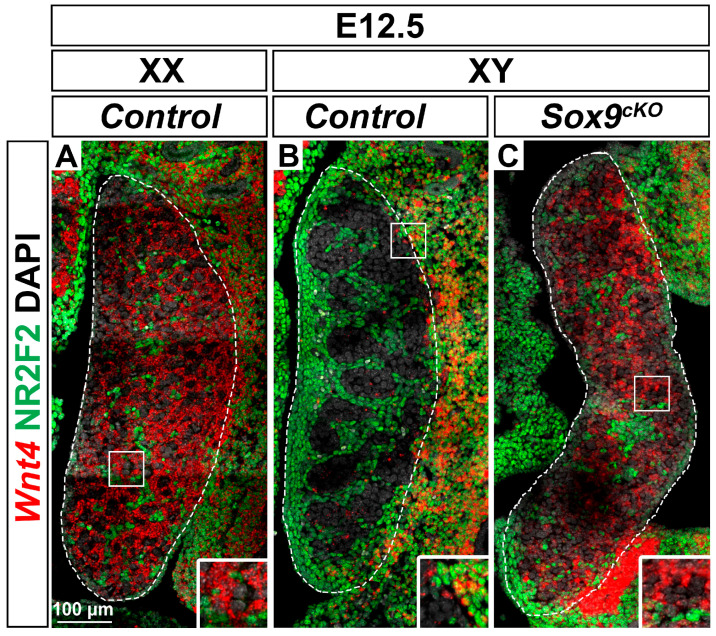
*Wnt4* expression is up-regulated in XY *Sox9* mutant gonads. (**A**,**B**) RNAscope^®^ detection of *Wnt4* transcripts (red) together with immunodetection of the interstitial and stromal cell marker NR2F2 (green) in E12.5 XX *Control* gonad (**A**), XY *Control* gonad (**B**), and XY *Sox9^cKO^* gonad (**C**). Nuclei labeled with DAPI are shown in grey. Magnification is the same in all panels. Scale bar = 100 μm. Gonads are outlined with broken white lines.

Next, we turned our attention to the role of *Wnt4* in XY *Sox9^cKO^* mutant sex reversal gonads developing as ovaries [11,12,13]. At E10.5, *Wnt4* is expressed in both XY and XX gonads [40] and is then down-regulated in developing testes at E11.5 [18,33,40,45,52,53]. In agreement, we detected abundant *Wnt4* transcripts in E12.5 ovaries while the expression was almost absent in stage-matched testes (Figure 3A,B). In the ovary, germ cells and NR2F2 positive stromal cells exhibited low levels of *Wnt4* transcripts (Figure 3A). In contrast, NR2F2 negative cells strongly expressed *Wnt4* (Figure 3A) indicating that *Wnt4* is predominantly expressed in pre-granulosa cells of the ovary [54,55]. These expression profiles were also observed in XY SOX9 loss-of-function gonads (Figure 3C), in agreement with the complete sex reversal reported in XY *Sox9^cKO^* mutants [11,12,13].

### 3.5. XY Supporting Cell Precursors Adopt a Female-Like Fate in Sox9^cKO^ Mutants Independently of WNT4

**Figure 4:**
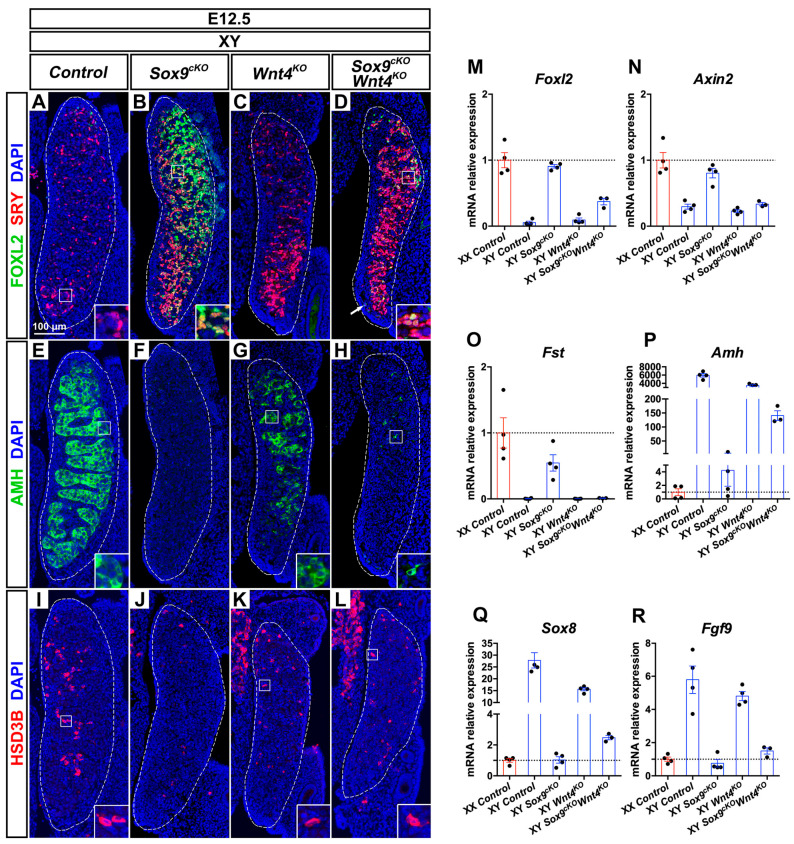
Phenotypic and gene expression changes in E12.5 XY *Sox9; Wnt4* mutants (**A**–**D**). Immunodetection of the pre-granulosa cell marker FOXL2 (green) and the male supporting cell marker SRY (red) in E12.5 XY gonads of *Control* (**A**), *Sox9^cKO^* (**B**), *Wnt4^KO^* (**C**), and *Sox9^cKO^ Wnt4^KO^* (**D**) genotypes. (**E**–**H**) Immunodetection of the Sertoli cell marker AMH (green) in E12.5 XY gonads of *Control* (**E**), *Sox9^cKO^* (**F**), *Wnt4^KO^* (**G**), and *Sox9^cKO^ Wnt4^KO^* (**H**) genotypes. (**I**–**L**) Immunodetection of the steroidogenic enzyme HSD3B (red) in E12.5 XY gonads of *Control* (**I**), *Sox9^cKO^* (**J**), *Wnt4^KO^* (**K**), and *Sox9^cKO^ Wnt4^KO^* (**L**) genotypes. (**M**–**R**) RT-quantitative PCR analysis of *Foxl2* (**M**), *Axin2* (**N**), *Follistatin* (*Fst*) (**O**), *Amh* (**P**), *Sox8* (**Q**), and *Fgf9* (**R**) expression in E12.5 gonads of XX *Control*, XY *Control*, XY *Sox9^cKO^*, XY *Wnt4^KO^*, and XY *Sox9^cKO^ Wnt4^KO^* genotypes (*N* = 3–4 embryos for each genotype). Expression level in XX controls is 1. Graphs show individual values (dots), and the mean fold-change (bars) ± SEM. Nuclei labeled with DAPI are shown in blue. Magnification is the same in all panels. Scale bar = 100 μm. Gonads are outlined with broken white lines. The white arrow in **D** indicates the coelomic vessel.

**Figure 5:**
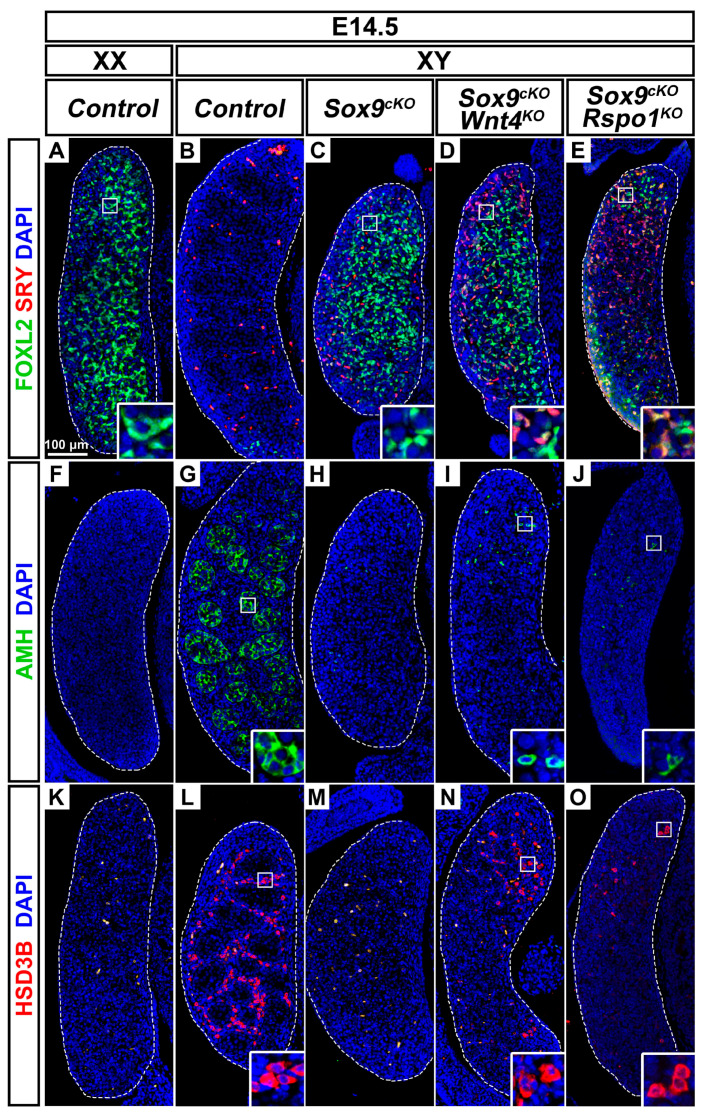
*Wnt4* or *Rspo1* deletion does not restore testicular development in embryonic XY gonads lacking SOX9 function. (**A**–**E**) Immunodetection of the pre-granulosa cell marker FOXL2 (green) and the male supporting cell marker SRY (red) in E14.5 gonads of XX *Control* (**A**), XY *Control* (**B**), XY *Sox9^cKO^* (**C**), *XY Sox9^cKO^ Wnt4^KO^* (**D**), and XY *Sox9^cKO^ Rspo1^KO^* (**E**) genotypes. (**F**–**J**) Immunodetection of the Sertoli cell marker AMH (green) in E14.5 gonads of XX *Control* (**F**), XY *Control* (**G**), XY *Sox9^cKO^* (**H**), *XY Sox9^cKO^ Wnt4^KO^* (**I**), and XY *Sox9^cKO^ Rspo1^KO^* (**J**) genotypes. (**K**–**O**) Immunodetection of the steroidogenic enzyme HSD3B (red) in E14.5 gonads of XX *Control* (**K**), XY *Control* (**L**), XY *Sox9^cKO^* (**M**), *XY Sox9^cKO^ Wnt4^KO^* (**N**), and XY *Sox9^cKO^ Rspo1^KO^* (**O**) genotypes. Nuclei labeled with DAPI are shown in blue. Magnification is the same in all panels. Scale bar = 100 μm. Gonads are outlined with broken white lines.

In order to investigate the contribution of WNT4 function to the XY *Sox9^cKO^* mutant phenotype, we analyzed XY single and double mutant gonads. In XY control gonads, SRY is expressed from E10.5 in pre-Sertoli cells, and is down-regulated by E12.5 upon testis differentiation (Figure 4A and Figure 5B) [6,56]. XY *Wnt4^KO^* mutant gonads following a testis fate exhibited sustained expression of SRY at E12.5 (Figure 4C). This was in agreement with delayed Sertoli cell differentiation in these mutants, which leads to transient defects in testis cords organization and size [19,57,58].

In E12.5 XY *Sox9^cKO^* mutants, SRY was also maintained at E12.5 (Figure 4B) and was down-regulated by E14.5 (Figure 5C). However, unlike XY control and XY *Wnt4^KO^* mutant gonads, SRY and FOXL2 were co-expressed in XY *Sox9^cKO^* mutants (Figure 4B and Figure 5C). This indicated that XY supporting cell precursors adopt a female-like pre-granulosa cell identity in the absence of SOX9 function. Similarly, XY *Sox9^cKO^ Wnt4^KO^* double mutant gonads harbored cells co-expressing SRY and FOXL2 at E12.5 and E14.5 (Figure 4D and Figure 5D), though *Foxl2* transcripts were reduced when compared with XY *Sox9^cKO^* mutants and XX controls (Figure 4M and Figure S3).

In our analyses, we noted that at E12.5 and E14.5, XY *Sox9^cKO^ Wnt4^KO^* double mutants showed increased expression of male supporting markers *Sox8*, *Dhh*, and *Amh*, when compared with XY *Sox9^cKO^* single mutants (Figure 4P,Q and Figure S3). However, the expression level of these markers was at least 10 times lower than in XY control or *Wnt4^KO^* mutant testes. In addition, AMH protein could only be detected in rare scattered cells in E12.5 and E14.5 double mutants (Figure 4H and Figure 5I), whereas it is expressed in Sertoli cells forming testis cords in XY control or Wnt4KO mutant testes (Figure 4E,G and Figure 5G). Thus, in summary, we find that XY Sox9cKO Wnt4KO double mutant supporting cell precursors can adopt a pre-granulosa cell-like identity even in the absence of WNT4 function. Furthermore, ablation of *Wnt4* cannot restore Sertoli cell development in embryonic XY gonads lacking SOX9 function.

### 3.6. Wnt4 Deletion Restores Coelomic Vessel and Steroidogenic Cell Development in XY Sox9^cKO^ Mutants

XY *Sox9^cKO^* mutants exhibit high expression of the WNT/β-catenin targets *Axin2* and *Fst* similar to XX controls (Figure 4N,O and Figure S3). In contrast, in XY double mutants, ablation of *Wnt4* results in down-regulation of *Axin2* and *Fst* to XY control levels at E12.5 and E14.5 (Figure 4N,O and Figure S3). Loss of *Fst* expression provides an explanation for the formation of a coelomic vessel in XY double mutants, as compared with XY *Sox9^cKO^* single mutants (Figure 4D, arrow) [30]. In addition, XY *Sox9^cKO^ Wnt4^KO^* double mutant gonads exhibited abundant HSD3β-positive steroidogenic cells (Figure 4L and Figure 5N), unlike XY *Sox9^cKO^* single mutants where only rare HSD3β-positive cells are observed (Figure 4F). These observations indicate that *Wnt4* deletion and decreased WNT/β-catenin signaling restore two testis-specific traits in XY *Sox9^cKO^* mutants, coelomic vessel formation, and steroidogenic cell development.

### 3.7. Pre-Granulosa-Like Cells are Specified in XY Sox9^cKO^ Rspo1^KO^ Double Mutants

Given that WNT4 is not involved in SRY and FOXL2 co-expression in XY *Sox9^cKO^* mutant supporting cells (Figure 4D), we asked whether RSPO1, an upstream factor of WNT4 in WNT/β-catenin signaling [18,21] would be required. Indeed, XY *Sox9^cKO^ Rspo1^KO^* double mutant mice exhibit hypo-plastic testes at post-natal stages, indicating that *Rspo1* deletion promotes testicular development in XY *Sox9^cKO^* mutants [46]. Compared with XY *Sox9^cKO^ Wnt4^KO^* gonads at E14.5, XY *Sox9^cKO^ Rspo1^KO^* mutant gonads exhibited fewer FOXL2-positive cells, with some co-expressing SRY (Figure 5D,E). This finding indicates that even in the absence of RSPO1 function, embryonic XY supporting cell precursors lacking SOX9 adopt a female-like identity. Like XY *Sox9^cKO^ Wnt4^KO^* double mutants, XY *Sox9^cKO^* mutants lacking *Rspo1* exhibited interspersed AMH-positive cells that were not organized in testis cords, and steroidogenic cells expressing HSD3β (Figure 5J,O).

We thus conclude that as in XY *Sox9^cKO^ Wnt4^KO^* mutants, ablation of *Rspo1* does not completely prevent the sex reversal in embryonic XY gonads lacking SOX9 function. Together with the observations in *Sox9^cKO^ Ctnnb1^cKO^* XY double mutants [26], our findings highlight that SOX9 function in the developing fetal testis extends beyond the inhibition of WNT/β-catenin signaling.

### 3.8. Pre-Granulosa-Like Cells are Not Maintained in XY Sox9^cKO^ Wnt4^KO^ Double Mutant Gonads

**Figure 6:**
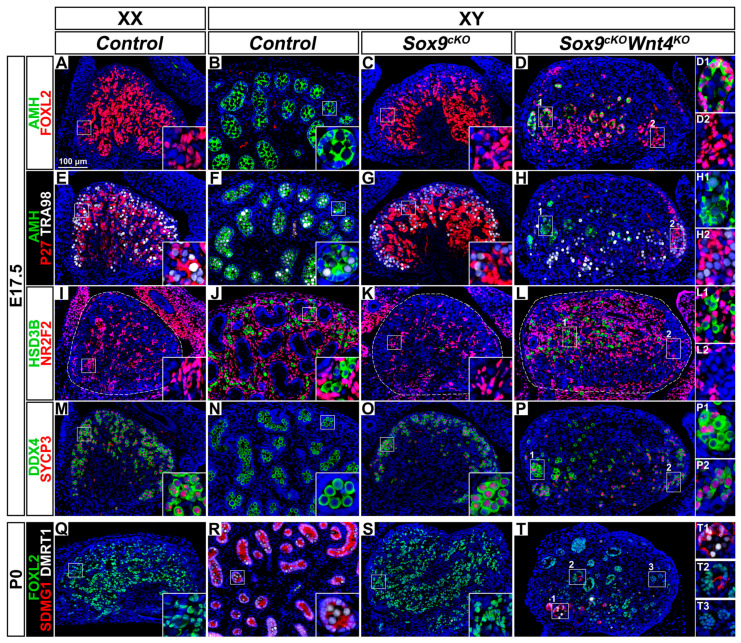
Ovotestis development in XY *Sox9; Wnt4* double mutant gonads at E17.5 and P0. (**A**–**D**) Immunodetection of the granulosa differentiation and Sertoli cell marker AMH (green) and the pre-granulosa cell marker FOXL2 (red) in E17.5 gonads of XX *Control* (**A**), XY *Control* (**B**), XY *Sox9^cKO^* (**C**), and *XY Sox9^cKO^ Wnt4^KO^* (**D**) genotypes. (**E**–**H**) Immunodetection of AMH (green), the quiescent cell marker P27 (red) and the germ cell marker TRA98 (white) in E17.5 gonads of XX *Control* (**E**), XY *Control* (**F**), XY *Sox9^cKO^* (**G**), and *XY Sox9^cKO^ Wnt4^KO^* (**H**) genotypes. (**I**–**L**) Immunodetection of the steroidogenic cell marker HSD3ß (green) and the interstitial and stromal cell marker NR2F2 (red) in E17.5 gonads of XX *Control* (**I**), XY *Control* (**J**), XY *Sox9^cKO^* (**K**), and *XY Sox9^cKO^ Wnt4^KO^* (**L**) genotypes. (**M**–**P**) Immunodetection of the germ cell marker DDX4 (green) and the meiosis marker SYCP3 (red) in E17.5 gonads of XX *Control* (**M**), XY *Control* (**N**), XY *Sox9^cKO^* (**O**), and *XY Sox9^cKO^ Wnt4^KO^* (**P**) genotypes. (**Q**–**T**) Immunodetection of the pre-granulosa cell marker FOXL2 (green), the Sertoli cell marker SDMG1 (red) and the Sertoli cell and male germ cell marker DMRT1 (white) in newborn gonads of XX *Control* (**Q**), XY *Control* (**R**), XY *Sox9^cKO^* (**S**), and *XY Sox9^cKO^ Wnt4^KO^* (**T**) genotypes. Nuclei labeled with DAPI are shown in blue. Magnification is the same in all panels. Scale bar = 100 μm. Regions marked by numbers in panels **D**, **H**, **L**, **P**, and **T** are shown in the corresponding insets. Gonads are outlined with broken white lines in **I**, **K**, and **L**.

To complete our analyses in XY *Sox9^cKO^ Wnt4^KO^* double mutant gonads, we examined gonads near birth, at E17.5. At this stage, XY *Sox9^cKO^* mutant gonads were indistinguishable from control ovaries with respect to the expression of FOXL2 and P27 in quiescent pre-granulosa cells throughout the gonad (compare Figure 6A with Figure 6C, and Figure 6E with Figure 6G) and the absence of steroidogenic cells (Figure 6I,K). Additionally, as in control fetal ovaries, germ cells in XY *Sox9^cKO^* mutant gonads initiate meiosis [11,12,13], as indicated by the expression of SYCP3, a meiotic marker (Figure 6O and Figure S4). In contrast, XY *Sox9^cKO^ Wnt4^KO^* mutant gonads displayed a mixed, part-ovary, part-testis, gonadal fate. The more posterior regions of the double mutant gonads exhibited fetal ovarian characteristics: FOXL2 and P27 expression (Figure 6D,H), absence of steroidogenic cells (Figure 6L), and presence of meiotic germ cells marked by the co-expression of DDX4 and SYCP3 (Figure 6P). In contrast, the anterior region exhibited hallmarks of ovary-to-testis reprogramming. This includes: down-regulation of P27 and up-regulation of AMH, indicating pre-granulosa cell loss of quiescence and pre-mature differentiation (Figure 6D,H); and as in control testes, steroidogenic cells expressing HSD3β and non-meiotic germ cells (Figure 6L,P). We also noted that mature granulosa cells co-expressing FOXL2 and AMH were found in tubular structures that resembled the organization of testis cords (Figure 6D).

Our results reveal that FOXL2 expressing pre-granulosa-like cells formed during the male-to-female sex reversal caused by *Sox9* mutation, fail to be maintained and prematurely differentiate in the anterior region of XY *Sox9^cKO^ Wnt4^KO^* double mutant gonads. This observation is in agreement with the known WNT4 function in the maintenance of pre-granulosa cell identity in XX gonads [25].

We next investigated whether Sertoli cells were formed in the anterior masculinized region of XY double mutants. DMRT1 is co-expressed with SDMG1 in Sertoli cells of control testes at P0, and is also found in germ cells inside the testis cords (Figure 6R) [51,59]. In XY double mutants, cells co-expressing DMRT1 and SDMG1 were observed, indicating that Sertoli cell differentiation had occurred (Figure 6T). Some tubular structures contained both FOXL2 positive and SDMG1 positive cells, suggesting that Sertoli-like cells trans-differentiate from granulosa cells in XY double mutants (Figure 6T).

Together these results indicate that XY *Sox9^cKO^ Wnt4^KO^* double mutant gonads develop as ovotestes near birth. In the anterior region, XY *Sox9^cKO^ Wnt4^KO^* mutant-supporting cells cannot maintain pre-granulosa cell identity in the absence of WNT4 function, and prematurely differentiate and trans-differentiate into Sertoli-like cells. We conclude that WNT4 function in XY *Sox9^cKO^* mutant gonads is dispensable for the specification of pre-granulosa cells, but is required for their maintenance.

## 4. Discussion

### 4.1. SOX9 Function is Dispensable for Partial Sex-Reversal in XX Wnt4^KO^ Mutants

Given that the testis-specific factor SOX9 is transiently up-regulated at E11.5 in XX *Wnt4^KO^* mutant gonads developing as ovotestes [40], it was assumed that SOX9 drives the partial sex reversal. However, we observed that in XX *Sox9^cKO^ Wnt4^KO^* double mutant gonads, pre-granulosa cells prematurely differentiate and testis-specific traits (coelomic vessel and steroidogenic cells) appear. These phenotypes are also present in XX gonads lacking *Wnt4*, *Rspo1*, *Ctnnb1*, and in XX *Sox9^cKO^* mutant gonads lacking *Rspo1* or *Ctnnb1* [18,19,20,21,25,26,46,60]. Thus, though required for male sex determination in XY gonads [11,12,13], SOX9 is dispensable for ovotestes development in XX gonads with impaired WNT/β-Catenin signaling.

In XX WNT/β-Catenin signaling single mutants, premature granulosa cell differentiation precedes reprogramming as Sertoli-like cells in embryonic gonads near or at birth [25,26,46,60]. In XX *Wnt4^KO^* single mutant gonads specifically, a previous report shows an absence of Sertoli-like cells expressing DMRT1 at E18.5 and presence of Sertoli-like cells expressing SOX9 (albeit sporadic) at birth [25]. In the present study, we did not detect Sertoli cells expressing DMRT1 in XX *Sox9^cKO^ Wnt4^KO^* mutants at E17.5 and P0. Note that analyses at post-natal stages in WNT4 loss-of-function mice were not possible, due to perinatal lethality [25,61]. Thus, by birth, XX *Sox9^cKO^ Wnt4^KO^* gonads lacked Sertoli-like cells that were present in XX *Sox9^cKO^* mutant gonads additionally lacking *Rspo1* or *Ctnnb1* [25,26]. The absence or delay of granulosa-to-Sertoli cell reprogramming in XX *Sox9^cKO^ Wnt4^KO^* mutant gonads is likely attributed to compensatory action of other WNT proteins to maintain pre-granulosa cells [25,26,60]. Alternatively, WNT4 function may specifically be required for aspects of Sertoli cell differentiation [57]. Altogether, our examination of XX *Sox9^cKO^ Wnt4^KO^* mutant gonads demonstrates that both SRY and SOX9 are dispensable for the loss of fetal ovarian integrity and the masculinization of XX *Wnt4^KO^* mutant gonads.

### 4.2. SRY Regulation in XY Sox9^cKO^ Wnt4^KO^ Double Mutants

Given that XY *Sox9^cKO^* mutant gonads develop as ovaries expressing *Wnt4* [11,12,13], we analyzed XY *Sox9^cKO^ Wnt4^KO^* double mutants to determine the contribution of WNT4 function in the XY sex reversal ovary.

We have observed that SRY expression is maintained in E12.5 XY *Sox9^cKO^* and *Sox9^cKO^ Wnt4^KO^* mutants, indicating that without the downstream activation of SOX9, SRY cannot antagonize granulosa cell formation to initiate Sertoli cell differentiation. SRY expression is down-regulated in control testes around E12.5 [6,56]. We found that SRY was widely detected in XY *Sox9^cKO^* mutants at E12.5 and was further maintained in E14.5 *Sox9^cKO^ Wnt4^KO^* and *Sox9^cKO^ Rspo1^KO^* mutants. It has been previously suggested that SOX9 might be involved in *Sry* down-regulation in the XY developing gonad [11]. However, additional factors are at play as demonstrated by the correct down-regulation of SRY expression at the poles of B6-XY^POS^ ovotestes devoid of SOX9 expression [62]. Interestingly, SRY expression is rescued in XY *Cbx2 Wnt4* mutant gonads [63], indicating that WNT4 function, possibly through the activation of β-catenin signaling, directly or indirectly contributes to a negative regulation of *Sry*.

### 4.3. Pre-Granulosa Cells are Specified in Sox9^cKO^ Mutants Without WNT4 Function

We show here that in the absence of WNT4 function, SRY-positive cells in XY *Sox9^cKO^ Wnt4^KO^* mutant gonads still adopt a pre-granulosa cell-like fate at E12.5, though *Foxl2* is expressed at reduced levels compared to XY *Sox9^cKO^* single mutants. Thus, WNT4 function is dispensable for early differentiation of XY *Sox9^cKO^* mutant supporting cells as pre-granulosa-like cells.

In XX gonads, granulosa cell specification and ovarian development require the WNT/β-Catenin and FOXL2 pathways acting in parallel. Ectopic up-regulation of β-Catenin signaling or FOXL2 in XY gonads has been shown to be sufficient to trigger granulosa cell differentiation [34,36]. In addition, the two pathways can compensate for each other as evidenced by (i) pre-granulosa cell specification before reprogramming as Sertoli-like cells in XX gonads lacking *Rspo1*, *Wnt4*, *Ctnnb1*, or *Foxl2*, [16,17,18,19,20,21,25,26] and (ii) enhancement of this phenotype in XX *Rspo1* or *Wnt4* mutants also carrying a *Foxl2* mutation [48,49]. In XY *Sox9^cKO^ Wnt4^KO^* mutant gonads, FOXL2 expression and reduced levels of WNT/β-Catenin signaling are sufficient for pre-granulosa-like cell specification from XY supporting cell precursors thus preventing embryonic testicular development. In contrast, mutations in *Wnt4* can rescue *Sox9* expression and testis development in XY embryos lacking FGF9 or its receptor FGFR2, indicating that WNT4 function antagonizes testis differentiation downstream of FGF9/FGFR2 signaling [45]. In *Fgf9 Wnt4* and *Fgfr2 Wnt4* double mutant gonads, the maintenance of SOX9 expression leads to the activation/reinforcement of a testis-differentiating pathway independent of FGF9/FGFR2 signaling [45]. This likely involves prostaglandin D2 signaling given that this pathway can activate *Sox9* expression independently of FGF signaling to promote Sertoli cell specification [64].

Altogether, our results further highlight that *Sox9* is indispensable to promote Sertoli cell specification at the time of sex determination.

### 4.4. RSPO1/WNT/β-Catenin Mutations Cannot Restore Sertoli Cell Specification in Sox9^cKO^ Mutants

The pre-granulosa-like cells observed at E14.5 both in XY *Sox9^cKO^ Wnt4^KO^* mutant gonads (this study) and in XY *Sox9^cKO^ Ctnnb1^cKO^* mutant gonads [26], prompted us to examine early XY *Sox9^cKO^ Rspo1^KO^* mutant gonad development. We previously reported post-natal hypo-plastic testis development with rare FOXL2 expressing follicles in XY *Sox9^cKO^ Rspo1^KO^* double mutants [46]. This raised the question of the early events leading to the hypo-plastic testis. We show here that at E14.5, XY *Sox9^cKO^ Rspo1^KO^* gonads contain SRY-positive cells also expressing FOXL2, indicating transient pre-granulosa cell-like specification. We presume that after initial pre-granulosa-like cell specification, these cells trans-differentiate as Sertoli-like cells, as in XX *Rspo1^KO^* single mutant gonads [25,46]. We conclude that SOX9 function is required for the early choice that drives XY Sertoli cell specification from a bipotential supporting cell precursor and that this function extends beyond the inhibition of RSPO1/WNT/β-Catenin signaling.

FOXL2-positive cells observed in XY *Sox9^cKO^* mutant gonads also harboring mutations in the WNT/β-Catenin pathway suggests that β-Catenin signaling is not required for *Foxl2* expression in these gonads. This may be explained by the loss of SOX9, which represses *Foxl2* expression in XY supporting cells either directly, or through the action of its target FGF9 [10,41]. Interestingly, ChIP-seq experiments have identified FOXL2 binding peaks in a region containing an enhancer involved in *Sox9* repression in granulosa cells [36]. Thus, FOXL2 and SOX9 might repress each other expression to ensure the appropriate differentiation of bipotential supporting precursors along the male or female pathway.

### 4.5. Vasculature Formation and Steroidogenic Cell Development Do Not Require SOX9 Function

In studying early gonad development in XY double mutants, it was apparent that inactivation of *Wnt4* in XY *Sox9^cKO^* mutant gonads restored a subset of testis-specific traits, including coelomic vessel formation and steroidogenic cell differentiation. The coelomic vessel forms in developing testes through the migration of endothelial cells of mesonephric origin [27]. This requires stimulation by Activin B, a homodimer with monomeric subunits (*Inhbb*) expressed by Sertoli and granulosa cells [65]. In developing ovaries, this process is inhibited by FST, an antagonist of Activin, a down-stream factor of the RSPO1/WNT/β-Catenin and FOXL2 pathways [18,20,21,30,31,36,66]. Reduced *Fst* expression in the absence of *Wnt4* in XY *Sox9^cKO^ Wnt4^KO^* double mutants is the most likely cause of coelomic vessel formation in these gonads.

With respect to steroidogenic cell differentiation in control developing testes, Hedgehog signaling (Desert Hedgehog, *Dhh*) is required for fetal Leydig cells differentiation from undifferentiated interstitial precursors [67]. *Dhh* expression is up-regulated in XY *Sox9^cKO^ Wnt4^KO^* double mutants compared to XY *Sox9^cKO^* mutants, suggesting that Hedgehog signaling might be involved in steroidogenic cell formation in these gonads.

Altogether our observations indicate that at least two male specific processes, coelomic vasculature and fetal steroidogenic cell formation, do not depend on SOX9 itself but require the inhibition of WNT signaling in XY gonads. In XX gonads lacking WNT4 function (XX *Wnt4^KO^* and XX *Sox9^cKO^ Wnt4^KO^* mutants), we also observed down-regulation of *Fst* and up-regulation of *Dhh* expression, when compared with control and XX *Sox9^cKO^* mutant ovaries, providing an explanation for the formation of male-like coelomic vessel and steroidogenic cells in these XX mutant gonads.

### 4.6. Premature Granulosa Cell Differentiation and Sertoli Trans-Differentiation in XY Sox9^cKO^ Wnt4^KO^ Double Mutants

In XY *Sox9^cKO^ Wnt4^KO^* double mutant gonads, a fraction of pre-granulosa cells exit mitotic arrest at E17.5 and begin to express AMH, indicating precocious granulosa cell differentiation. We conclude that the maintenance of pre-granulosa cell identity in XY *Sox9^cKO^* mutant ovaries requires WNT4 function. Premature granulosa cell differentiation is a common feature of XX gonads lacking *Rspo1*, *Wnt4* or *Ctnnb1* [25,26]. This process is independent of SOX9 function as shown here in XX *Sox9^cKO^ Wnt4^KO^* double mutants, but also in XX *Sox9^cKO^ Rspo1^KO^* and XX *Sox9^cKO^ Ctnnb1^cKO^* double mutant gonads [26,46]. Currently, it is unknown whether premature granulosa cell differentiation is a cell autonomous process triggered by the decrease of β-Catenin signaling or linked to other testis-like phenotypes such as coelomic vasculature and steroidogenic cells.

It is noteworthy that premature granulosa cell differentiation in XY double mutant gonads occurs predominantly in the anterior region of the gonad. This is similar to the situation observed in XX *Sox9^cKO^ Wnt4^KO^*, XX *Sox9^cKO^ Rspo1^KO^*, XX *Sox9^cKO^ Ctnnb1^cKO^* double mutants and XX *Wnt4^KO^*, XX *Rspo1^KO^* and XX *Ctnnb1^cKO^* single mutants [25,26,46]. Thus, it appears that premature granulosa cell differentiation in XX and XY ovotestes proceeds along an anterior-to-posterior axis that recapitulates the gradient of differentiation observed in normal ovarian development [68].

We observed testis cord-like structures with Sertoli-like cells co-expressing DMRT1 and SDMG1 in newborn XY *Sox9^cKO^ Wnt4^KO^* mutant gonads. These structures contained granulosa cells expressing FOXL2, suggesting that the Sertoli-like cells originate from reprogramming of granulosa cells. We did not observe Sertoli-like cells expressing DMRT1 in XX double mutants, indicating that the Y chromosome, and possibly some target genes of SRY [26], favor Sertoli cell development in XY *Sox9^cKO^ Wnt4^KO^* ovotestes.

Altogether, we report that Sertoli-like cells in XY *Sox9^cKO^ Wnt4^KO^* mutant gonads arise from a two-step process: differentiation of XY pre-granulosa cells due to loss of SOX9 function, then premature granulosa cell differentiation followed by Sertoli cell reprogramming, due to loss of WNT4 function.

## 5. Conclusions

This work demonstrates that SOX9 and WNT/β-Catenin signaling act at different levels in the mutual antagonistic network that drives supporting cell differentiation and gonadal development. SOX9 function is essential for the initial decision in the supporting cell lineage to drive Sertoli cell differentiation. WNT/β-Catenin signaling co-operates with FOXL2 for granulosa cell specification, inhibits male vascular and steroidogenic development and is essential to maintain pre-granulosa cells in an undifferentiated quiescent state during ovarian development.

## Supplementary Materials

The following are available online at https://www.mdpi.com/2073-4409/9/5/1103/s1, Figure S1: Sox9 expression in control and mutant gonads at E12.5 and E14.5; Figure S2: Phenotype of E14.5 XX control and mutant gonads; Figure S3: Quantitative PCR analysis of gene expression in XY control and mutant gonads at E14.5; Figure S4: Germ cell development in XY control and mutant gonads at E14.5; Table S1: Antibodies for immunodetection; Table S2: Quantitative-PCR primers.
