# Supplementary material for: Mouse Gonad Development in the Absence of the Pro-Ovary Factor WNT4 and the Pro-Testis Factor SOX9
Source: Cells. 2020 Apr 29;9(5):1103. doi: 10.3390/cells9051103 (PMC7291083; doi:10.3390/cells9051103)
Supplement: Supplementary file 1 [file cells-09-01103-s001.pdf]

cells MDPI

Mouse gonad development in the absence of the pro-ovarian factor WNT4 and the pro-testis factor SOX9

Furong Tang[1], Nainoa Richardson[1], Audrey Albina[1], Marie-Christine Chaboissier[1,#] and Aitana Perea-Gomez[1,#,*]

Supplementary Materials

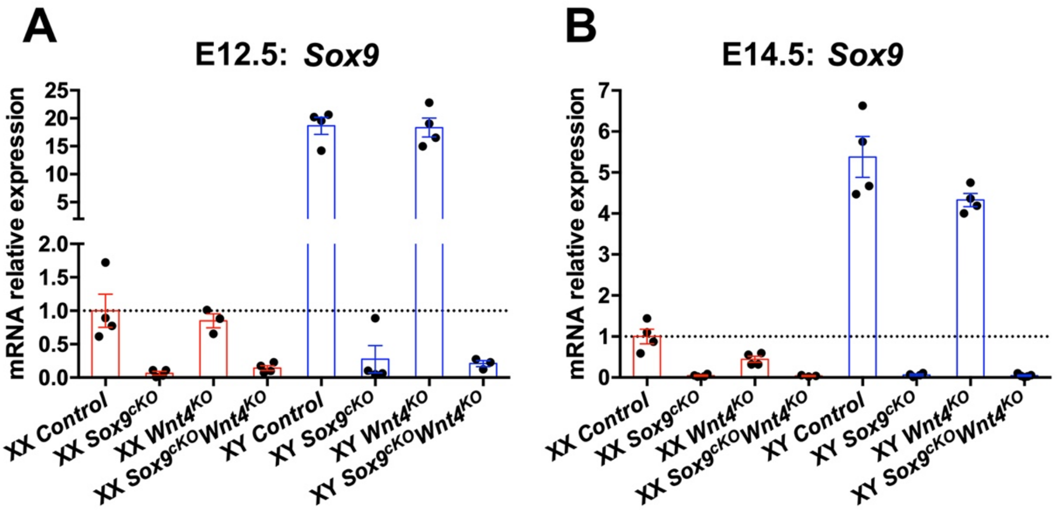

Figure S1. *Sox9* expression in control and mutant gonads at E12.5 and E14.5. RT-quantitative PCR analysis of *Sox9* expression in E12.5 (A) and E14.5 (B) XX and XY gonads of *Control*, *Sox9^{cKO}*, *Wnt^{4KO}* and *Sox9^{cKO}Wnt4^{KO}* genotypes (n = 3 to 5 embryos for each genotype). Expression level in XX controls is 1. Graphs show individual values of the relative normalized expression levels (dots), and the mean fold-change (bars) ± SEM.

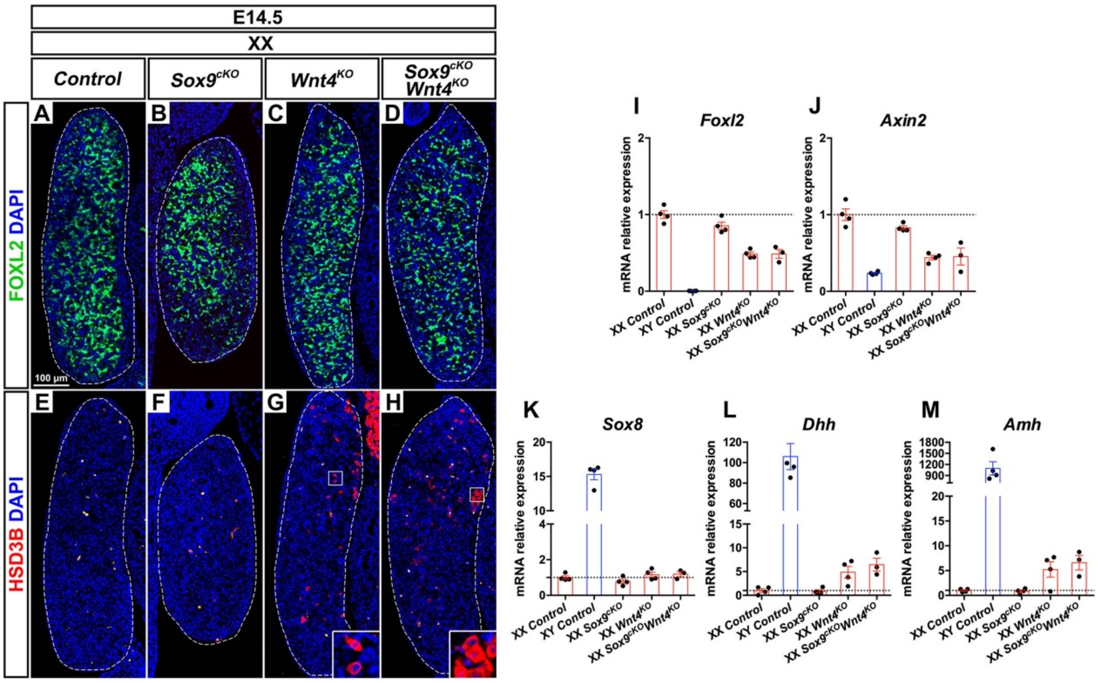

Figure S2. Phenotype of E14.5 XX control and mutant gonads. (A-D) Immunodetection of the pre-granulosa cell marker FOXL2 (green) in E14.5 XX gonads of *Control* (A), *Sox9*^{cKO} (B), *Wnt4*^{KO} (C) and *Sox9*^{cKO}*Wnt4*^{KO} (D) genotypes. **(E-H)** Immunodetection of the steroidogenic enzyme HSD3B (red) in E14.5 XX gonads of *Control* (E), *Sox9*^{cKO} (F), *Wnt4*^{KO} (G) and *Sox9*^{cKO}*Wnt4*^{KO} (H) genotypes. **(I-M)** RT-quantitative PCR analysis of *Foxl2* (I), *Axin2* (J), *Sox8* (K), *Dhh* (L) and *Amh* (M) expression in E14.5 gonads of XX *Control*, XY *Control*, XX *Sox9*^{cKO}, XX *Wnt4*^{KO} and XX *Sox9*^{cKO}*Wnt4*^{KO} genotypes (n = 3 to 4 embryos for each genotype). Expression level in XX controls is 1. Graphs show individual values (dots), and the mean fold-change (bars) ± SEM. Nuclei labeled with DAPI are shown in blue. Magnification is the same in all panels. Scale bar = 100 µm. Gonads are outlined with broken white lines.

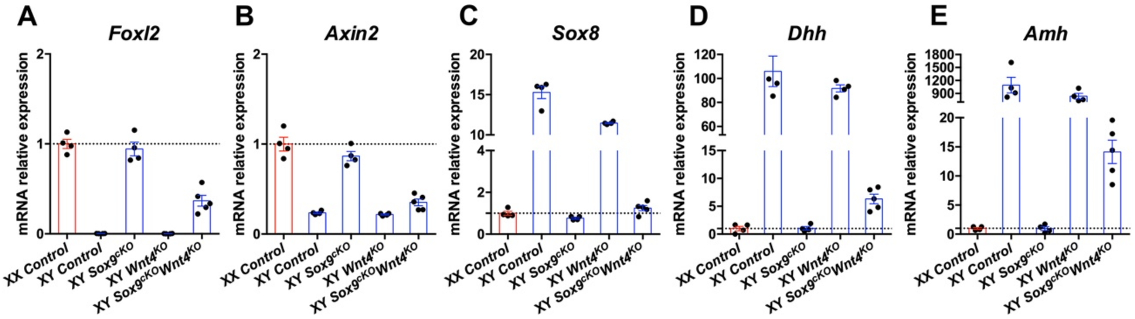

Figure S3. Quantitative PCR analysis of gene expression in XY control and mutant gonads at E14.5. RT-quantitative PCR analysis of *Foxl2* (A), *Axin2* (B), *Sox8* (C), *Dhh* (D) and *Amh* (E) expression in E14.5 gonads of XX *Control*, XY *Control*, XY *Sox9^{cKO}*, XY *Wnt^{4KO}* and XY *Sox9^{cKO}Wnt4^{KO}* genotypes (n = 4 to 5 embryos for each genotype). Expression level in XX controls is 1. Graphs show individual values of the relative normalized expression levels (dots), and the mean fold-change (bars) ± SEM.

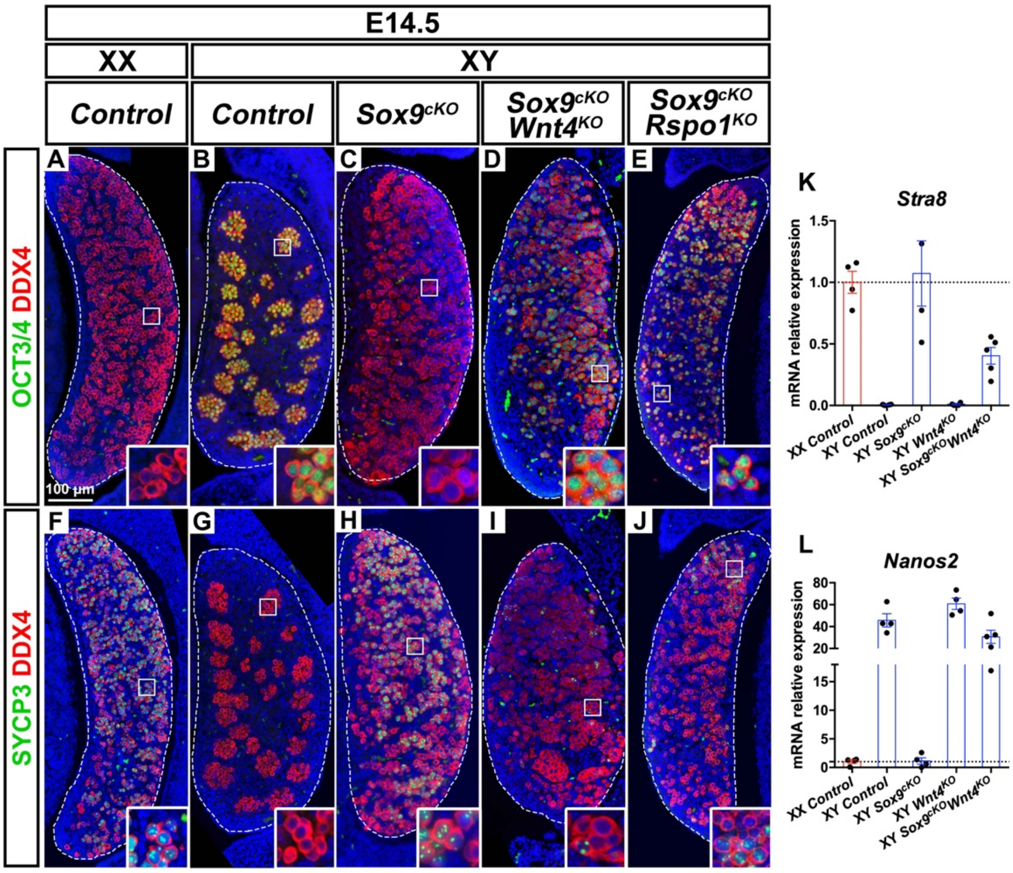

Figure S4. Germ cell development in XY control and mutant gonads at E14.5. (A-E) Immunodetection of germ cell marker DDX4 (red) and the pluripotent cell marker OCT3/4 (green) in E14.5 gonads of XX *Control* (A), XY *Control* (B), XY $Sox9^{cKO}$ (C), *XY* $Sox9^{cKO}Wnt4^{KO}$ (D) and XY $Sox9^{cKO}Rspo1^{KO}$ (E) genotypes. **(F-J)** Immunodetection of germ cell marker DDX4 (red) and the meiosis marker SYCP3 (green) in E14.5 gonads of XX *Control* (F), XY *Control* (G), XY $Sox9^{cKO}$ (H), *XY* $Sox9^{cKO}Wnt4^{KO}$ (I) and XY $Sox9^{cKO}Rspo1^{KO}$ (J) genotypes. The majority of germ cells in *XY* $Sox9^{cKO}Wnt4^{KO}$ and XY $Sox9^{cKO}Rspo1^{KO}$ express OCT3/4 like in XY controls. Only a few meiotic germ cells expressing SYCP3 can be observed at this stage in double mutant gonads. **(K-L)** RT-quantitative PCR analysis of the meiosis marker *Stra8* (K) and the male germ cell marker *Nanos2* (L) in E14.5 gonads of XX *Control*, XY *Control*, XY $Sox9^{cKO}$, XY $Wnt4^{KO}$ and XY $Sox9^{cKO}Wnt4^{KO}$ genotypes (n = 4 to 5 embryos for each genotype). Expression level in XX controls is 1. Graphs show individual values of the relative normalized expression levels (dots), and the mean fold-change (bars) ± SEM. Nuclei labeled with DAPI are shown in blue. Magnification is the same in all panels. Scale bar = 100 µm. Gonads are outlined with broken white lines.

Table S1: Antibodies for immunodetection.

Antibody	Species	Supplier	Reference	Dilution
**DDX4**	Rabbit	Abcam	ab13840	200X
**TRA98**	Rat	Abcam	82527	200X
**AMH**	Mouse	Bio-Rad Laboratories	MCA2246	30X
**AMH**	Goat	Santa Cruz Biotechnology	sc6886	200X
**OCT3/4**	Mouse	BD Transduction Laboratories	611202	200X
**FOXL2**	Goat	Novus Biologicals	NB100-1277SS	200X
**NR2F2**	Mouse	R&D Systems	PP-H7147-00	200X
**SOX9**	Rabbit	Merck KGaA	HPA001758	250X
**P27**	Rabbit	Santa Cruz Biotechnology	sc-52	200X
**DMRT1**	Mouse	Santa Cruz Biotechnology	SC-377167	200X
**HSD3B**	Goat	Santa Cruz Biotechnology	sc-30820	200X
**SYCP3**	Mouse	Santa Cruz Biotechnology	Sc-74569	200X
**SRY**	Rabbit	Dagmar Wilhelm's Lab		200X
**SDMG1**	Rabbit	Ian Adams's Lab		600X

Table S2: quantitative-PCR primers.

Gene	Forward 5' - 3'	Reverse 5' - 3'
Axin2	GCAAGTCCAAGCCCCATA	CGGCTGACTCGTTCTCCT
Foxl2	ATCGGGGTTCCTCAACAAC	CATCTGGCAGGAGGCGTA
Fst	GCCTATGAGGGAAAGTGTATCAA	TGGAATCCCATAGGCATTTT
Sox9	GCGGAGCTCAGCAAGACTCTG	ATCGGGGTGGTCTTTCTTGTG
Sox8	GACCCTAGGCAAGCTGTGG	CTGCACACGGAGCCTCTC
Fgf9	TGCAGGACTGGATTTCATTTAG	CCAGGCCCACTGCTATACTG
Dhh	GGACCTCGTACCCAACTACAA	CGATGGCTAGAGCGTTCACC
Amh	GGGGAGACTGGAGAACAGC	AGAGCTCGGGCTCCCATA
Nanos2	AGGTCCCCCGATCTCAAC	CAGCATTTCCCAGTGTTTCAG
Stra8	TGACGTGGCAAGTTTCCTG	GTTGCAGGTGGCAAACATAG
